# Prenatal care and infant outcomes of teenage births: a Project WATCH study

**DOI:** 10.1186/s12884-023-05662-x

**Published:** 2023-05-24

**Authors:** Madelin E. Gardner, Amna Umer, Toni Rudisill, Brian Hendricks, Candice Lefeber, Collin John, Christa Lilly

**Affiliations:** 1 Department of Epidemiology/Biostatistics, School of Public Health, West Virginia University, SPH 64 Medical Center Dr, PO Box 9190, Morgantown, WV 26505 USA; 2 Department of Pediatrics, School of Medicine, West Virginia University, Morgantown, WV USA

**Keywords:** Prenatal care, Teen pregnancy, APGAR, NICU

## Abstract

**Introduction:**

Infants of teenage births are known to have increased risk of poor infant outcomes. Adequate prenatal care (PNC) is essential to the overall health of infants and their birthing persons. While teenage births continue to be of concern in rural areas, little is known about the association between inadequate PNC and poor infant outcomes in teenage populations.

**Purpose:**

To determine the association between inadequate PNC (< 10 visits) and poor infant outcomes neonatal intensive care unit (NICU) stay, low APGAR score, small for gestational age (SGA) and length of stay (LOS).

**Methods:**

The study used West Virginia (WV) Project WATCH population level data (May 2018-March 2022). Multiple logistic regressions and survival analysis were performed on infant outcomes; NICU stay, APGAR score, infant size, and infant length of stay (LOS) with PNC categories inadequate (< 10 PNC visits) vs adequate (10 or more) adjusting for covariates including maternal race, insurance status, parity, smoking status, substance use status, and diabetes status.

**Results:**

Of births to teenagers, 14% received inadequate PNC. Teens who received inadequate PNC had increased odds of infant admitted to NICU (aOR: 1.84, CI:(1.41, 2.42), *p* < 0.0001), low 5- minute APGAR score (aOR: 3.26, CI:(2.03,5.22), *p* < 0.0001), and increased LOS (Est. = -0.33, HR: 0.72, CI:(0.65,0.81), *p* < 0.0001).

**Conclusions:**

Results demonstrated that infants of teenagers who received inadequate PNC are at increased risk of requiring a NICU stay, having a low APGAR score and requiring an increased LOS. PNC is particularly important for these groups as they are at increased risk of poor birth outcomes.

## Background

Pregnancy and birth complications are the number one cause of death of girls ages 15–19 globally [1, 2]. Approximately 21 million teenagers give birth each year [1, 2]. While global rates of teenage pregnancy have been decreasing since the 1970’s, in developed nations, such as the US, Canada, and Western Europe, teenage pregnancy rates remain high [3]. In developing countries, these adolescent pregnancies are more likely to be planned and within the union of marriage [4]. In developed counties however, adolescent pregnancies are typically unplanned and occur in unmarried women [4]. In addition to maternal mortality, teenage pregnancy increases the risk of infant mortality, preterm birth, low birth weight, placental abruption, and eclampsia [1, 2, 5, 6]. Teenage births are also associated with decreased education or educational prospects, decreased work prospects, menarche at early age, lack of sexual education, and family history of teenage births [7]. Teenage birthing persons are at increased risk of living in poverty, being excluded by their peers and community, and have increased barriers to education post-birth [7]. For this study teenage births is defined as a live infant born to an individual less than 20 years of age.

### Prenatal care (PNC)

One medical necessity that may mitigate some of these poor health profiles includes patient-physician interactions during prenatal care (PNC) visits. PNC is essential to the health of mother and baby [8–14], as it gives clinicians the opportunity to advise young mothers and to prepare them for birth and motherhood. These interactions with health care providers can include exchanging pregnancy and birth information, facilitation of education, screening measures for abnormalities and complications for mother and baby, monitoring/continuous care, and preparation for childbirth and motherhood [12, 13]. PNC also provides the possibility of early detection and possible treatment of diseases, initiate timely intervention, promote overall wellness, and aid in facilitating informed birth choices [8, 14].

The most common and publicly well-validated indices for adequate PNC are the Kessner Index and the Adequacy of Prenatal Care Utilization (APNCU) index (or Kotelchuck Index). The Kessner Index classifies PNC into three categories: adequate, intermediate, and inadequate. For PNC to be considered adequate, initiation must begin in the first trimester and there must be 9 or more visits total for a pregnancy of 36 weeks or more [15]. The Adequacy of Prenatal Care Utilization (APNCU) index (or Kotelchuck Index) uses two parameters, including time of initiation and number of visits [16]. The APNCU index bases the number of visits off of the ACOG standards for uncomplicated healthy pregnancies that is used by practicing physicians as PNC standard of medical care [16]. This index categorizes care into four categories: inadequate, intermediate, adequate, and adequate plus. Adequate PNC is 80–109% of expected PNC visits, or 9 to 13 total visits [16–18]. Both indices require initiation of care in the first trimester.

### Infant outcomes of teenage births

There are many adverse infant outcomes related to teenage births already known in the literature. Teen pregnancy has been linked with preterm birth and low birthweight babies [2, 6, 19]. While much literature has observed the association between poor infant outcomes and teenage births, the relationship is confounded by social and economic conditions [2]. Pregnant teenagers are more likely to be poverty-stricken, of minority racial or ethnic status, have less education, and be unmarried compared to their older peers [7, 20–22].

### Teenage births and PNC

While little is known about the relationship between PNC inadequacy and adverse infant outcomes of teenage births, one recent study showed that teenage mothers who received inadequate PNC (RR: 1.82 (95% CI: 1.39, 2.37)) and intermediate PNC (RR: 1.58 (95% CI: 1.83, 2.57)) were at increased risk of maternal morbidity when compared to teenage mothers who received adequate PNC [5]. The study also found that teenage mothers with maternal comorbidities who received inadequate PNC had 5 times increased risk of maternal mortality than those without maternal comorbidities and adequate PNC [5].

### West Virginia

West Virginia (WV) is one of the poorest states economically and in terms of population health. According to the WV Behavioral Risk Factor Surveillance System (BRFSS), WV is ranked number 2 nationally for the highest prevalence of adults who report being in fair or poor health [23]. The prevalence of no healthcare coverage in the state is 14.9%, compared to only 10.1% nationally, and one fifth of adults do not have a personal healthcare provider (PCP) [23]. WV also has a very high rate of teenage births with 22.5 per 1,000 births being from a teenager aged 15–19 in 2020 [24] compared to the national average for 2020 of 15.3 per 1,000 [24].

In summary, there is a strong relationship between teen births and poor infant outcomes. Additionally, there is strong relationship between inadequate PNC and poor infant outcomes, while little is known about the relationships between teen births and inadequate PNC. To our knowledge no other study has examined infant outcomes of teen births directly in a statewide analysis. Our study aims to fill these gaps and examine the association of inadequate PNC and infant outcomes among teenage population giving birth in WV. We hypothesize that teenage mothers who do not receive inadequate PNC will have infants who are smaller for gestational age, lower APGAR scores, more likely to go to NICU, and are more likely to have longer hospital stays than infants of teenage mothers who receive adequate PNC. This is hypothesized because PNC is designed to monitor mother and fetus for possible complications that could arise over the course of the pregnancy. Without appropriate monitoring and evaluation complications that arise could go untreated leading to poorer infant outcomes.

## Methods

This study used data from the Project WATCH/WV Birth Score Program. This dataset collects surveillance data on all infants and their pregnant persons born in WV. This is a unique dataset to WV fully funded by WV Division of Health and Human Resources. This dataset provides additional information not found on birth certificate data that allows the state to identify infants who are at greatest risk for poor health and care, and has made significant contributions in the reduction of mortality in infants from 1 month to 1 year of age [25]. The dataset has a 98–99% match to available birth certificate data, and relative to this study, additional variables such as substance use and APGAR score. The proposed study used data from the years May 2018 – March 2022 resulting in a total sample of 70,724 individuals; the data was subset to include all live hospital births to teenagers (< 20 years old) (*n* = 4,347) 6.2% of total sample. The datasets generated and/or analyzed during this study are not publicly available due to funding agreements, but aggerate datasets are available from the corresponding author on reasonable request.

### Independent variable

The exposure variable for this study is inadequacy of PNC. Inadequacy of PNC is measured as a binary variable defined as inadequate care as < 10 PNC visits and adequate care a as ≥ 10 PNC visits. While there are many ways to define inadequate PNC, this method was chosen due to a previous study using this dataset by Umer et al. which determined by way of Receiver Operating Curve (ROC) analysis that 10 or 11 PNC visits optimized the sensitivity and specificity for increased risk of infant mortality, and also determined that the strength of the bivariate associations were stronger with < 10 PNC visits being the cut off for inadequate PNC [26]. This definition of inadequate PNC is consistent with both the Kessner and APNCU indices.

### Dependent variables

The main outcome variables of interest were length of infant hospital stay (LOS), small for gestational age (SGA), infant stay in the Neonatal Intensive Care Unit (NICU) and APGAR score. APGAR score is a method for assessing an infant after birth. Elements in the APGAR score assessment include; color of infant, heart rate, reflexes, muscle tone, and respiration rate [27]. These variables of interest are based on previous literature [2, 6, 19] and information that is collected and available within the dataset. Infant stay in NICU, APGAR score and SGA were analyzed as binary variables. Infant stay in NICU was binary (yes vs no) and captures if the infant was admitted to the NICU, including those transferred to a NICU at a different hospital. For this study 5-min APGAR score that ranges from 0 – 10 was recoded into low APGAR score being less than 7 and normal being 7 or greater this cutoff value is based on literature [27]. Using data on birthweight (grams) and gestational age (weeks), gestational age categories were computed. Small for gestational age (SGA) was defined as infants born with a birth weight below the 10th percentile, appropriate for gestational age (AGA) defined as infants born with a birth weight between the 10th percentile and the 90th percentile, and large for gestational age (LGA) was defined as infants born with a birth weight above the 90th percentile. These cut-off values were based on recommendations by the World Health Organization’s (WHO) [28]. AGA and LGA were combined in this study due to both groups having very similar PNC rates. LOS was analyzed as a continuous count variable of days in the hospital, where infant discharge date was subtracted for date of birth. In this dataset the discharge date is captured on final hospital discharge so even if an infant was transferred to a different unit or hospital, the discharge date captured is the final discharge date.

### Covariates

Socio-demographic and confounding variables were controlled for in this analysis, including maternal race, education, parity, insurance payment method, smoking status, diabetes, substance use, and maternal age. Project WATCH collects data on race as a categorical variable with categories white, Black, Asian, Hispanic, Multiracial, and other. For this study, race was dichotomized into white vs not white. Maternal age was gathered as a continuous data ranging from 11 to 51 years old. Data was subset to only include teen births aged 19 and under. Maternal education was collected as a continuous variable and was recategorized for the purpose of demographics into 8^th^ grade or less, 9^th^ grade, 10^th^ grade, 11the grade 12^th^ grade, and some college. Parity included number of previous pregnancies and was collected as a continuous variable. Parity was then categorized as 0, 1, 2, and 3 or more for demographic purpose but was then recoded into 0 and 1 or more for simplicity in the model. For this study, insurance status was originally collected as private insurance, WV Medicaid, self-pay, other, and unknown was reclassified into private insurance vs other. Smoking was collected as nicotine use during pregnancies and was based on self-report data. Smoking status was categorized as yes vs no. Substance use data includes opioids, sedatives/hypnotics, cannabinoids, alcohol, stimulants, phencyclidine- PCP, gabapentin, and antidepressants and was based on either self-report, prenatal records, and drug test of birthing person, data categorized as yes vs no. Diabetes was collected as type I, type II, gestational diabetes, or none and was recategorized to any diabetes and no diabetes. Maternal age and education were highly correlated (*r* = 0.60, < 0.0001), so only maternal age was included in the models.

### Statistical analysis

All statistical analysis was conducted in SAS version 9.4 (SAS Institute, Cary NC). Missing data was treated using pairwise deletion. Basic descriptive statistics were performed on all variables. Frequencies and percentages were calculated for adequacy of PNC groups in teen births categorical demographic characteristics, and covariates for the full sample, and then stratified by adequate/inadequate PNC. Chi-square tests were performed with accompanying *p*-values presented to determine the significance of the associations between covariates and PNC. Means and standard deviations were calculated for continuous variables. Bivariate associations for continuous variables included t-tests or Mann–Whitney tests to evaluate their relationship with PNC. Logistic regression analysis was used to examine the bivariate relationship between PNC and each categorical health outcome, small for gestational age (SGA), NICU, and low APGAR score, with covariates. Covariates were binary coded for the logistic regression analysis, including payment method (WV Medicaid vs other), Race (white vs not white), Parity (0 vs 1 or more), smoking (no vs yes), substance use (no vs yes), diabetes (no vs yes), while maternal age was kept continuous. Results are presented as odds ratio (OR) and adjusted OR (aOR) along with 95% confidence intervals (CI). Kaplan–Meier curves were used to determine if the probabilities of LOS differed between PNC groups, and a Weibull model was used to perform a survival analysis to determine the bivariate relationship between PNC groups and the continuous count outcome, infant length of stay (LOS). A Weibull model was selected due to ties, appropriate model shape, and having the lowest AIC value [29]. Adjusted and unadjusted hazard ratios (HR) and 95% confidence intervals (CI) along with *p*-values and regression coefficients were calculated for having inadequate PNC; this type of model was selected due to high number of ties (e.g., 2 day stays) among a large proportion of the sample. All covariates were adjusted for in the final model. Since prematurity is in the causal pathway to LOS, a post hoc sensitivity analysis was conducted to determine that appropriateness of the model by stratifying by term and pre-term births, and comparing the resulting HRs.

## Results

The study population (*n* = 4,347) was predominately white (i.e., 92.1%), 62% had at least a 12^th^ grade education, 75% of the population had no previous pregnancies, 69% had WV Medicaid insurance, 79% were non-smokers, and 87% did not use substance during pregnancy (need to define this variable in the method section). Of the total population, 7% of the infants born were of small for gestational age, 2% had an APGAR score of less than 7, and 9% of infants required a stay in the NICU. Of all births to teenagers, 14% received inadequate PNC. Other descriptive statistics and differences by adequate v. inadequate PNC are provided in Table 1.

**Table 1:** Study characteristics of all teenagers who gave birth to infants in WV (*n* = 4,347)

Variables	Total Frequency(Percent)	Inadequate PNC Frequency(Percent)	Adequate PNC Frequency(Percent)	*P*-value
**Race**				0.3033
White	3950(92.1%)	534(13.5%)	3416 (86.5%)	
Black	131(3.1%)	18(13.7%)	113 (86.3%)	
Hispanic	36(0.8%)	7(19.4%)	29 (86.6%)	
Multiracial	106(2.5%)	19(17.9%)	87 (82.1%)	
Other	65(1.5%	13(20.0%)	52 (80.0%)	
**Maternal Education**				< 0.0001
8th Grade or Less	99(2.3%)	22(22.2%)	77 (77.8%)	
9th Grade	258(5.9%)	54(20.9%)	204 (79.1%)	
10th Grade	450(10.4%)	85(18.9%)	365 (81.1%)	
11th Grade	845(19.5%)	133(15.7%)	712 (84.3%)	
12th Grade	2245(51.7%)	271(12.1%)	1974 (87.9%)	
Some College	445(10.3%)	37(8.3%)	408 (91.7%)	
**Parity**				0.0443
0	3273(75.3%)	427(13.0%)	2846 (87.0%)	
1	820(18.9%)	131(16.0%)	689 (84.0%)	
2	195(4.5%)	35(17.9%)	160 (82.1%)	
3 or more	59(1.4%)	10(16.9%)	49 (83.1%)	
**Payment Method**				0.0726
WV Medicaid	2988(68.7%)	431(14.4%)	2557 (85.6%)	
Private	847(19.5%)	95(11.2%)	752 (88.8%)	
Self-Pay	34(0.8%)	8(25.5%)	26 (76.5%)	
Other	412(9.5%)	59(14.3%)	353 (85.7%)	
Unknown	66(1.5%)	10(15.2%)	56 (84.9%)	
**Smoking Status**				< 0.0001
Yes	919(21.1%)	175(19.0%)	744 (81.0%)	
No	3427(78.9%)	427(12.5%)	3000 (87.5%)	
**Substance Use**				< 0.0001
Yes	582(13.4%)	139(23.9%)	443 (76.1%)	
No	3765(86.6%)	464(12.3%)	3301 (87.7%)	
**Conditions**				0.0265
No diabetes	4162(96.6%)	584(14.0%)	3578 (86.0%)	
Type I Diabetes	12(0.3%)	2(16.7%)	10 (83.3%)	
Type II Diabetes	7(0.2%)	1(14.3%)	6 (85.7%)	
Gestational Diabetes	128(3.0%)	6(4.7%)	122(95.3%)	
**Infant Size**				0.3924
Small for Gestational Age	311(7.2%)	51(16.4%)	260(83.6%)	
Average for Gestational Age	1953(44.9%)	264(13.5%)	1689(86.5%)	
Large for Gestational Age	2083(47.9%)	288(13.8%)	1795(86.2%)	
**APGAR Score**				< 0.0001
Less than 7	85(2.0%)	29(34.1%)	56(65.9%)	
7 or Greater	4262(98%)	574(13.5%)	3688(86.5%)	
**Infant Stay in NICU**				< 0.0001
Yes	378(8.7%)	86(22.8%)	292(77.2%)	
No	3969(91.3%)	517(13.0%)	3452(87.0%)	
	**Mean(SD)**	**Mean(SD)**	**Mean(SD)**	
**Length of Stay**	3.15(8.70)	4.37(14.74)	2.94(7.15)	
**Maternal Age**	18.15(1.14)	18.0(1.34)	18.18(1.10)	

^*^Column percentages used for total frequency and row percentages used for PNC group

### Infant outcomes: NICU, APGAR, and SGA

For all births to teens in WV during the study period, compared to teenagers who received adequate PNC, the odds of an infant admitted to NICU was significantly increased when inadequate PNC was received during the pregnancy (Table 2; aOR: 1.84, CI:(1.41, 2.42), *p *< 0.0001). The odds of infants having a low 5- minute APGAR score when teenagers received inadequate PNC were significantly increased (Table 2; aOR: 3.26, CI:(2.03,5.22), *p* < 0.0001). Increased odds were found for infants being SGA when born to teens who received inadequate PNC compared to those who received adequate PNC, those results were not statistically significant (Table 2; aOR: 1.08, CI:(0.78, 1.50), *p* = 0.6302).

**Table 2:** Unadjusted and adjusted odds ratios of infant outcomes NICU admission, APGAR score, and SGA by PNC (*n* = 4,347)

**Dependent Variable**			**Odds Ratio (95% CI)**	**Chi-Square**	***P*-Value**
**NICU Admission**	**Unadjusted Model**	**Prenatal Care**			
		Less than 10	1.967(1.520,2.544)	1642.5	< 0.0001
		10 or more	1		
	**Adjusted Model**	**Prenatal Care**			
		Less than 10			
		10 or more	1.84(1.41,2.42)	19.5	< 0.0001
	**Covariates**	**Payment Method**			
		WV Medicaid	1		
		Other	0.95(0.750,1.21)	0.16	0.6932
		**Race**			
		White	1		
		Non-White	1.10(0.75,1.61)	0.19	0.6635
		**Parity**			
		0	1		
		1 or more	0.98(0.76,1.27)	0.03	0.8716
		**Smoking**			
		No	1		
		Yes	0.91(0.69,1.20)	0.49	0.4842
		**Substance Use**			
		No	1		
		Yes	1.42(1.05,1.91)	5.15	0.0232
		**Diabetes**			
		No	1		
		Yes	2.46(1.57,3.85)	15.42	< 0.0001
**5- minute APGAR score less than 7**	**Unadjusted Model**	**Prenatal Care**			0.2469
		Less than 10	3.33(2.107,5.255)	967.3	< 0.0001
		10 or more	1		
	**Adjusted Model**	**Prenatal Care**			
		Less than 10	3.26(2.03,5.22)	23.98	< 0.0001
		10 or more	1		
		**Payment Method**			
		WV Medicaid	1		
		Other	0.87(0.53, 1.42)	0.32	0.5696
		**Race**			
		White	1		
		Non-White	1.35(0.66,2.76)	0.68	0.4088
		**Parity**			
		0	1		
		1 or more	1.12(0.66,1.84)	0.18	0.6677
		**Smoking**			
		No	1		
		Yes	0.90(0.52,1.58)	0.12	0.724
		**Substance Use**			
		No	1		
		Yes	1.09(0.59,2.03)	0.08	0.7754
		**Diabetes**			
		No	1		
		Yes	2.13(0.84,5.39)	2.54	0.1107
**Infant Small for Gestational Age**	**Unadjusted Model**	**Prenatal Care**			0.5415
		Less than 10	1.24(0.91,1.70)	1.79	0.1814
		10 or more	1		
	**Adjusted Model**	**Prenatal Care**			
		Less than 10	1.08(0.78,0.150)	0.23	0.6302
		10 or more	1		
	**Covariates**	**Payment Method**			
		WV Medicaid	1		
		Other	1.20(0.94,1.55)	2.05	0.1521
		**Race**			
		White	1		
		Non-White	1.39(0.93,2.06)	2.6	0.1069
		**Parity**			
		0	1		
		1 or more	0.93(0.70,1.22)	0.31	0.5785
		**Smoking**			
		No	1		
		Yes	1.98(1.52,2.58)	25.5	< 0.0001
		**Substance Use**			
		No	1		
		Yes	1.55(1.14,2.11)	7.97	0.0048
		**Diabetes**			
		No	1		
		Yes	0.45(0.18,1.11)	3.03	0.0816
		**Age of Mother**	1.04(0.93,1.17)	0.55	0.4591

An exploration of significant confounders also revealed interesting information about teenage births and PNC. Individuals who had diabetes (Type I, II or gestational) were almost 2.5 times greater odds (aOR: 2.46, CI:(1.57,3.85), *p* < 0.0001) to have an infant admitted to the NICU. It was also found that teenagers who smoke were at twice the odds (aOR: 1.98, CI:(1.52,2.58), *p* < 0.0001) and teenagers that used substances were 1.5 times greater odds (aOR: 1.55, CI:(1.14,2.11), *p* = 0.0048) to have an infant classified as SGA.

### Infant outcome: LOS

**Fig. 1:**
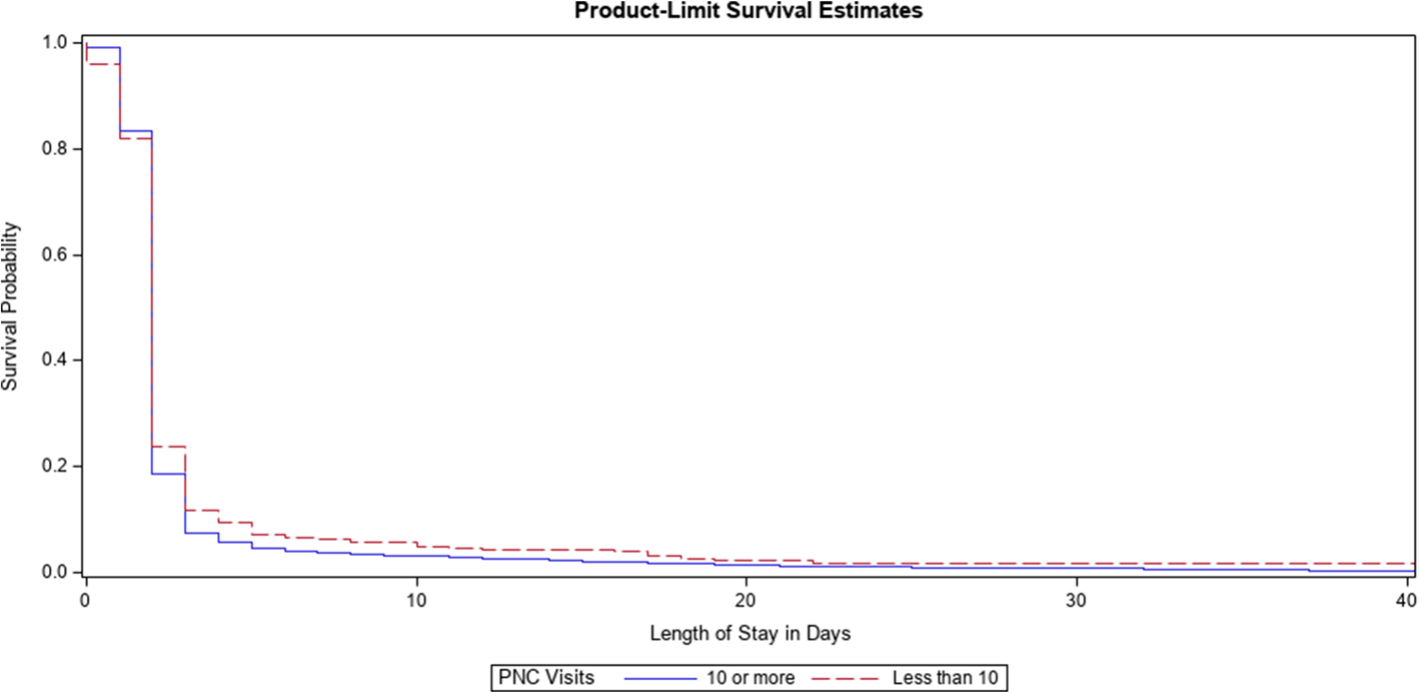
Survival analysis for infant length of stay in hospital stratified by teenagers who received inadequate vs. adequate PNC

Results of the Kaplan–Meier analysis show statistically different probabilities for LOS between teens who received inadequate and adequate PNC -2log(LR) test (χ2 = 58.72, *p* =  < 0.0001) LOS being longer for infants of teens who received inadequate PNC (mean stay: 4.37 days (SD = 14.74) compared to 2.95 days (SD = 7.15) Shown in Fig. 1.

The Weibull estimates, HRs, and their corresponding 95% CI and *p*-values are presented in Table 3. The results of the survival analysis show that infants of teenagers that received inadequate PNC had longer LOS compared to infants of teenagers that received adequate PNC (Est. = -0.33, HR: 0.72, CI:(0.65,0.81), *p* < 0.0001). The analysis also found that infants of non-white teenagers (Est. = -0.4, HR: 0.67, CI:(0.58,0.77), *p* < 0.0001) and infants of younger teenagers (Est. = -0, HR: 0.91, CI:(0.88,0.91), *p* < 0.0001) also had longer median LOS compared to their counterparts.

**Table 3:** Results of Weibull Survival Analysis for infant LOS by PNC for infants born to teenagers (*n* = 4,347)

**Dependent Variable**			**Regression Coefficient**	**Hazard Ratio (95% CI)**	**Chi-Square**	***P*-Value**
**Length of Stay**	**Unadjusted Model**	**Prenatal Care**				
		Less than 10	-0.41	0.66(0.59,0.74)	52	< 0.0001
		10 or more				
	Adjusted Model	**Prenatal Care**				
		Less than 10	-0.33	0.72(0.65,0.81)	32.62	< 0.0001
		10 or more				
	Covariates	**Payment Method**				
		WV Medicaid				
		Other	-0.01	0.99(0.91,1.07)	0.1	0.7521
		**Race**				
		White				
		Non-White	-0.4	0.67(0.58,0.77)	29.93	< 0.0001
		**Parity**				
		0				
		1 or more	-0.07	0.93(0.85,1.02)	2.36	0.1248
		**Smoking**				
		No				
		Yes	0.01	1.01(0.92,1.11	0.04	0.8448
		**Substance Use**				
		No				
		Yes	0.04	1.04(0.93,1.17)	0.44	0.5094
		**Diabetes**				
		No				
		Yes	-0.14	0.87(0.70,1.07)	1.73	0.1878
		**Age of Mother**	-0.09	0.91(0.88,0.94)	25.3	< 0.0001

The results of the post hoc sensitivity analysis stratifying by term and pre-term birth showed a slightly attenuated but similar hazard ratio estimate for term birth infants (HR:0.76, CI:(0.70,0.83). For pre-term infants, the hazard ratio was slightly more attenuated but still in the correct direction (HR:0.70, CI:(0.48,1.03) and a drop in significance (*p* = 0.06); suggesting this model may be best suited for term infants.

## Discussion

This study adds to the limited extant literature on infant outcomes of teenage births and inadequate PNC use in a rural Appalachian state of WV. WV has a very high rate of teenage births, with 22.5 per 1,000 births being from a teenager in 2020 [24] compared to the national average for 2020 of 15.3 per 1,000 [30]. The results show that 14% of teenage births in WV received inadequate PNC based off the definition of inadequate PNC determined by Umer et al. [26]. This statistic is in line with the national average of inadequate PNC across all age groups of approximately 15% in 2020 [14]; small differences could be attributed to differences in the measuring of inadequate PNC.

Prior literature concluded that infants of teenagers are at increased risk of poor infant outcomes [31, 32]. The results of this study conclude that infants of teenagers who receive inadequate PNC are at subsequent increased risk of certain poor infant outcomes, including SGA, NICU stay, longer LOS, and lower APGAR scores.

More specifically, the results of this study determined that a relationship exists between teenagers who receive inadequate PNC and their infants being born with low 5-min APGAR score (< 7). This is of particular concern, as the literature demonstrates that infants born with low APGAR scores have poor long-term cognitive outcomes including lower IQ scores and lower test scores at ages 15–16 [33].

Similarly, studies have found that having an infant requiring a NICU stay can have a negative impact on the overall mental health of the parents; these studies note elevated levels of anxiety and depression found in parents of NICU babies when compared to parents whose babies did not require a NICU admission [31, 32, 34]. This anxiety and depression can cause discomfort in the parent-infant interactions [34]. Substance use was also found to have a significant association with infants requiring admission to the NICU. This is in line with previous literature that has linked maternal substance use to premature births, smaller infant weight and length, and smaller head circumference [35]. Literature also reports that infants of substance-using birthing persons require longer hospital stays and NICU admission due to neonatal abstinence syndrome (NAS) and family dysfunction [35, 36].

While infant size was not found to be associated with inadequate PNC, covariates found to be significant were consistent with previous literature. For example, this study also noted a relationship between smoking and having an infant born SGA [37–39]. Infants SGA have been found to have significantly lower academic achievement later in life when compared to infants who were not SGA [40, 41].

Increased infant LOS was also found to be associated with inadequate PNC; while this relationship could be highly confounded with other factors such as medical conditions, the health of the mother, and characteristics of the mother-infant dyad, it is still worth noting. Longer infant LOS has been associated with mental distress on the parents of the infant, as well as strain on parent-infant bonding [34].

This study analyzes the risks of receiving inadequate PNC for infants of teenage pregnancies in the rural state of WV. Many poor infant outcomes can be attributed to inadequate PNC and other factors over the course of the pregnancy. PNC and other factors such as smoking, substance use, having diabetes, and being non-white have an impact on infant outcomes. Improving use of PNC on a state level could be a primary prevention measure in improving mother and infant health. While there is minimal research on interventions to improve PNC, there have been a few studies that have determined that incentives such as cash or baby items (i.e. car seats, baby blankets, etc.) have shown to improve overall PNC [42]. This, however, is not a feasible intervention due to costs within this population. This study concludes that inadequate PNC in teenagers is a risk factor for poor infant outcomes at time of delivery with long-term implications. Some additional barriers to PNC in rural populations include distance to the nearest clinic and available transportation [43]. These barriers could be addressed by increased access to public transportation and increased presence of clinicians within rural communities. We recommend that the strong association of poor infant outcomes, with teens that do not receive proper PNC in WV should be addressed by directing more targeted research and/or prevention measures aimed at improving education and access to PNC.

Teenagers experience additional barriers to PNC such as cost, fear, and lack of education. A secondary prevention measure to address the poor infant outcomes in teenagers would be to provide proper sex education and birth control methods to teenagers. Sexual education is more scrutinized and less common in rural school systems due to greater religious and community influence [44]. While most sexually active teens use some form of birth control (~ 90%), the most common forms used are condoms or birth control pills which are not the most effective and require consistent and correct use to prevent pregnancy [45]. Long-acting reversible contraception (LARC) methods such as intrauterine devices and implants are the most effective and could be a better option for teens [45].

### Limitations

There are limitations to this analysis, one of the most pertinent is the lack of information on a potential confounder of financial support within the household. There is a known association of poverty and teen pregnancy, and poverty and inadequate PNC [7, 20–22]; not including this known confounder might mean the implications of the study are indirectly due to SES rather than directly inadequate PNC. However, the inclusion of medical insurance status in the model may help to mitigate some of this potential bias. Similarly, there are many unknown health conditions of both the infant and mother that could have an association with infant outcomes in which this dataset does not account for, one in particular being maternal hypertension, while maternal hypertension is known to be a common morbidity in pregnancy and childbirth it is not collected in this dataset in the future we hope to be able to explore the association of inadequate PNC and infant outcomes while including maternal hypertension as a covariate. We were limited to de-identified data, because of this we are unable to account for births to multiples (twins, triplets, etc.) in this analysis. The lack of an ethnicity variable creates a limitation, Hispanic is considered a race in the race variable which doesn’t permit us to analysis ethnicity directly. While the state is largely non-Hispanic future work should include analyzing a state with more ethnic diversity. This is also a cross-sectional analysis; therefore, causal inferences cannot be drawn as temporality could not be assessed. The results of this study may not be generalizable to populations outside of WV. While not generalizable, the results of the study could potentially lead further research on a wider scale to determine association between LOS, infant size, and low APGAR score and PNC among teenage pregnancies other states or on a national level. Finally, the definition of PNC was previously defined in the Project WATCH dataset and could not be adjusted to fit other indexes of PNC [15, 46]. Despite these limitations, this study demonstrates the potential harm inadequate PNC can have on infant outcomes such as LOS, SGA, and low APGAR scores among teenage pregnancies in the state of WV.

## Conclusions

This analysis concludes the strong association of poor infant outcomes, including longer LOS, SGA, NICU stays, and low APGAR scores with teen births that do not receive proper PNC in WV. This association should be addressed by directing more targeted research and/or prevention measures aimed at improving education and access to PNC. While this study analyzed teenage births in WV we believe that the same barrier to PNC exist nationally and more research needs to be done to determine the most effective interventions to mitigate these barriers and improve the adequacy of PNC. This information contributes to the broader literature base as well; specifically, to literature on teenagers and their likelihood to receive PNC and the impact on infant outcomes.

## Data Availability

The datasets generated and/or analyzed during the current study are not publicly available due to funding agreements, but aggerate datasets are available from the corresponding author on reasonable request.

## Abbreviations

AGA: Average for gestational age
BRFSS: Behavior
CI: Confidence intervals
LARC: Long-acting reversible contraception
LGA: Large for gestational age
LOS: Length of stay
NAS: Neonatal abstinence syndrome
NICU: Neonatal intensive care unit
PCP: Personal healthcare provider
PNC: Prenatal care
SGA: Small for gestational age
WHO: World Health Organization
WV: West Virginia
